# 3D PHOVIS: 3D photoacoustic visualization studio

**DOI:** 10.1016/j.pacs.2020.100168

**Published:** 2020-03-10

**Authors:** Seonghee Cho, Jinwoo Baik, Ravi Managuli, Chulhong Kim

**Affiliations:** a School of Interdisciplinary Bioscience and Bioengineering, Pohang University of Science and Technology (POSTECH), Pohang, 37673, Republic of Korea; b Department of Creative IT Engineering, Pohang University of Science and Technology (POSTECH), Pohang, 37673, Republic of Korea; c Department of Bioengineering, University of Washington, Seattle, WA, 98195, USA; d Hitachi Healthcare America, Twinsburg, OH, 44087, USA; e Departments of Creative IT Engineering, Mechanical Engineering, Electrical Engineering, and School of Interdisciplinary Bioscience and Bioengineering, Pohang University of Science and Technology (POSTECH), Pohang, 37673, Republic of Korea

**Keywords:** Volume mosaic, 3D scan-conversion, Skin detection, Visualization, Rendering, Volume imaging, 3D imaging

## Abstract

Photoacoustic (PA) imaging (or optoacoustic imaging) is a novel biomedical imaging method in biological and medical research. This modality performs morphological, functional, and molecular imaging with and without labels in both microscopic and deep tissue imaging domains. A variety of innovations have enhanced 3D PA imaging performance and thus has opened new opportunities in preclinical and clinical imaging. However, the 3D visualization tools for PA images remains a challenge. There are several commercially available software packages to visualize the generated 3D PA images. They are generally expensive, and their features are not optimized for 3D visualization of PA images. Here, we demonstrate a specialized 3D visualization software package, namely 3D Photoacoustic Visualization Studio (3D PHOVIS), specifically targeting photoacoustic data, image, and visualization processes. To support the research environment for visualization and fast processing, we incorporated 3D PHOVIS onto the MATLAB with graphical user interface and developed multi-core graphics processing unit modules for fast processing. The 3D PHOVIS includes following modules: (1) a mosaic volume generator, (2) a scan converter for optical scanning photoacoustic microscopy, (3) a skin profile estimator and depth encoder, (4) a multiplanar viewer with a navigation map, and (5) a volume renderer with a movie maker. This paper discusses the algorithms present in the software package and demonstrates their functions. In addition, the applicability of this software to ultrasound imaging and optical coherence tomography is also investigated. User manuals and application files for 3D PHOVIS are available for free on the website (www.boa-lab.com). Core functions of 3D PHOVIS are developed as a result of a summer class at POSTECH, “High-Performance Algorithm in CPU/GPU/DSP, and Computer Architecture.” We believe our 3D PHOVIS provides a unique tool to PA imaging researchers, expedites its growth, and attracts broad interests in a wide range of studies.

## 1 Introduction

Photoacoustic imaging (PAI, or optoacoustic imaging) is a biomedical imaging technique that detects ultrasound (US) signals generated via light-induced thermal expansion and relaxation of cells within tissues, called the photoacoustic (PA) effect. [1] Based on the fundamental hybrid nature, PAI has two unique competitive differentiators: (1) strong image contrast on the basis of rich intrinsic (e.g., melanin [2], DNA/RNA [3], hemoglobin [[4], [5], [6]], lipid [7], water [8], and others [9]) and extrinsic (e.g., various contrast agents) optical properties, and (2) deep tissue imaging capability in an optical diffusion regime providing high spatial resolution [10]. Due to these unique features, not only biological applications but also medical applications have been investigated in the recent decade [[11], [12], [13], [14], [15]].

Many research activities in the PAI field boost the hardware and software performance, develop various novel PA derivative techniques, and apply to the technology in life science [[16], [17], [18]] and medicine [[19], [20], [21], [22], [23]]. However, research activities benefitting the visualization of the 3D PAI are rarely found. Mainly, few commercial medical data processing and visualization tools such as ImageJ (Wayne Rasband, USA), AMIRA (Thermo Fisher Scientific, USA) and VolView (Kitware, USA) are available, and they do not provide visualization tools specifically targeted for PAI. In addition, they are relatively expensive, do not support incorporating newly available visualization techniques, often have insufficient image qualities, and cannot be integrated with the most popular data processing tool in academia, i.e., MATLAB (MathWorks, USA).

**Fig. 1:**
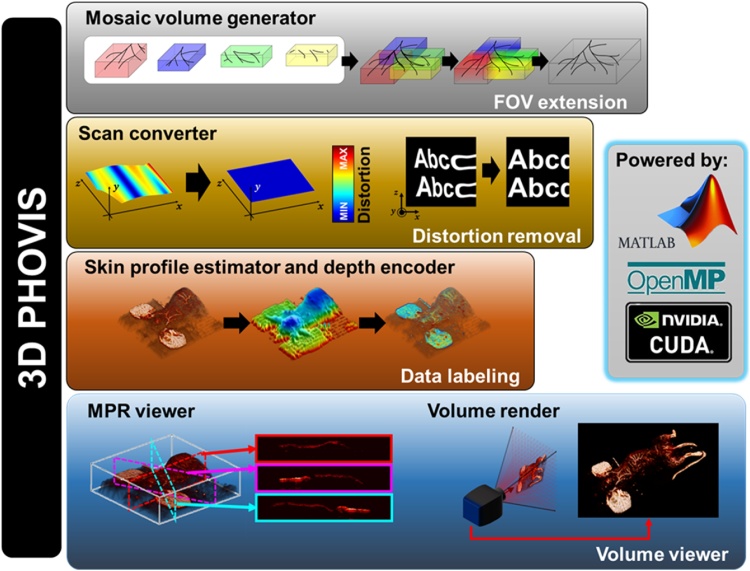
Main features of 3D Photoacoustic Visualization Studio (3D PHOVIS). FOV, field of view; PAM, photoacoustic microscopy; and MPR: multiplanar reformation.

Here, we have developed a novel 3D data/image processing and visualization tool for PAI, called 3D Photoacoustic Visualization Studio (3D PHOVIS). The unique features of 3D PHOVIS are illustrated in Fig. 1. We implemented the 3D PHOVIS on the most commonly used MATLAB platform and leveraged parallel processing technology to provide real-time interactive work interface. The MATLAB platform allows researchers to incorporate their own newly developed visualization algorithm to improve the image quality further. The 3D PHOVIS consist of the following applications: (1) a mosaic volume generator, (2) a scan converter for optical scanning photoacoustic microscopy (PAM), (3) a skin profile estimator and depth encoder, (4) a multiplanar reconstruction (MPR) viewer with a navigation map, and (5) a volume renderer with a movie maker. In addition to PAI, we also explored the use of the 3D PHOVIS for other popular biological and medical imaging tools, such as optical coherence tomography (OCT) and US imaging. We expect this free software package available on the website (www.boa-lab.com) will broadly impact the research of PAI in the field of biological and medical imaging.

## 2 Design concept

### 2.1 Goal of development and expected user environments

The goal of our 3D PHOVIS is to provide intuitive volume editing and high-quality volume visualization experiences for all researchers in the field of biomedical imaging. The 3D PHOVIS can handle any data type that can be imported into MATLAB. Detailed data preparation procedures are described in the instruction manual with sample scripts.

### 2.2 Preparation of test data set

Acoustic-resolution (AR) PAM system (MicroPhotoAcoustics, USA) with a water-immersible micro-electro-mechanical systems (MEMS) scanner (OPTICHO, Republic of Korea) was used to acquire 3D PAM volume data for operational testing. The animal experiments were performed as per regulations and protocols approved by the Institutional Animal Care and Use Committee (IACUC) of Pohang University of Science and Technology (POSTECH), and regulations of the National Institutes of Health Guide for the Care and Use of Laboratory Animals. We performed PAI of a healthy mouse to acquire 3D volumetric PAM data (POSTECH Biotech Center; 15−25 g, 6 weeks old) in a back-coronal view. We first anesthetized the mouse with a vaporized isoflurane (1 L/min of oxygen and 0.75 % isoflurane), and then removed its hair with depilatory lotion. The mouse was placed on an imaging table custom-designed for PAI during the *in vivo* imaging experiments and the mouse body temperature was maintained with a heating pad. Ultrasound gel (Ecosonic, SANIPIA, Republic of Korea) was applied to enhance acoustic impedance matching between the mouse body and the water. The optical energy illuminated on the mouse skin surface was 2.3 mJ/cm^2^, which is below the maximum permissible exposure for single laser pulse (MPE_SLP_) safety limits of 20 mJ/cm^2^ at 532 nm. For multiple pulse trains, the maximum permissible exposure for pulse train (MPE_train_) is 269 mJ/cm^2^, which is calculated for the scanning range of 2.5 mm, the optical beam size of 1.5 mm, the imaging pixel step size of 16.7 μm, and the exposure time of 3.6 ms. For 90 adjacent overlapped laser pulses, resulting maximum permissible exposure for MPE_SLP_ is 3.0 mJ/cm^2^, which is well above the illuminated optical energy of 2.3 mJ/cm^2^ in the imaging experiment [24]. The MPE_SLP_ and MPE_train_ are governed by American National Standards Institute (ANSI).

For demonstrating the use of 3D PHOVIS for OCT image rendering, we used the OCT data published by Golabbakhsh et al. [25]. For the US image rendering demo, we acquired a 3D US volume data of a fetus phantom (Model 065-36, CISR Inc., Norfolk, VA) with a mechanically-swiveling 3D transducer interfaced to the EUB-6500 ultrasound machine (Hitachi Healthcare America, USA).

### 2.3 Design of interactive graphical user interface

All graphical user interface (GUI) applications are developed using the 'GUIDE' toolkit provided by MATLAB 2016a. The GUI controllers are custom-developed to give a more intuitive experience than MATLAB built-in GUI controls. All applications utilized the custom-made slider classes because the default MATLAB slider class could not provide an interactive response when moving the slider. A custom-made color map editor class is implemented in every application and can be embedded in a GUI window. In addition, several custom-made interactive graphics classes are also developed to facilitate data visualization, such as navigation cursor, volume position tool, Gaussian brush tool, window/level tool, transfer function adjustment tool, and rendering direction setting tool.

### 2.4 Software structure

Since the interpreter-based software is slow, the functions based on MATLAB script are only used to determine the sequential processes or the interactive behavior of the GUI. If a function requires a two-dimensional loop process for calculation, we use a MATLAB built-in pre-compiled or custom-developed MATLAB executable (MEX) function. All loops in the MEX function are parallelized by the OpenMP library or streaming single instruction multiple data extensions (SSE). For functions requiring 3D data manipulation, we implemented parallel thread execution (PTX) kernels based on CUDA general-purpose computing for graphics processing unit (GPGPU). A majority of computation is implemented based on single-precision floating-point values for speed. All included functions and class structures are loaded by the main window of 3D PHOVIS. All applications are called in the main window and are associated with their GUIs and built-in features.

## 3 Result

### 3.1 Mosaic volume generator

#### 3.1.1 Background

One of the most efficient ways to create wide-field images is to mosaic sequentially acquired multiple images. Many techniques have been developed to create panoramic images in 2D by employing feature extraction algorithms on 2D dense data [26]. However, in many applications, automatic feature extraction and matching algorithms do not always work [27,28]. Thus, commercial 2D viewer usually support manual image alignment interfaces to flexibly position the individual images [27,29,30]. For PAI also, it is difficult to apply feature extraction algorithms because the data is sparse and three dimensional [31]. Thus, convenient user interface is necessary to precisely align the individual images to create a mosaic 3D volume imaging. We have prepared a manual volume positioning interface for the volume mosaic process, allowing users to simultaneously observe and assign 3D positions of volumes in multiple planes.

#### 3.1.2 Algorithm

**Fig. 2:**
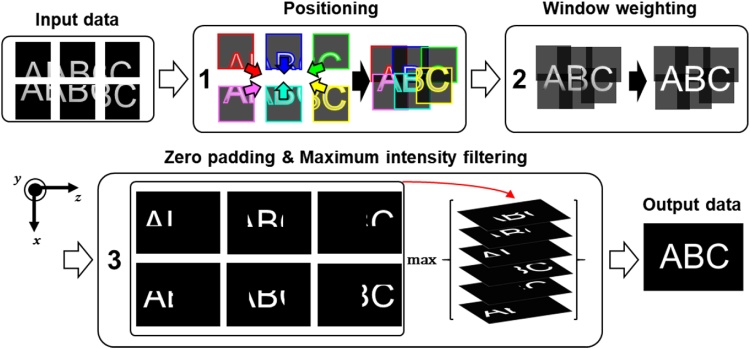
Schematic of a 3D volumetric mosaic process.

The volume merging algorithm consists of the following three steps: positioning, window weighting, and zero padding & maximum intensity filtering (Fig. 2). Note that all these steps are processed in the 3D domain. The first step to create a mosaic image is to identify the relative positions between individual images. In the proposed algorithm, the 3D positioning of each volumetric image set is determined manually. The difference in the PA signals in the overlapped regions are interpolated with window weighting to eliminate motion/ghost artifacts and compensate for uneven signal [28]. In the final step, zero-padding is applied to each volume data to match the size of the final volume, and the final volume is generated via maximum value filtering.

#### 3.1.3 Application

**Fig. 3:**
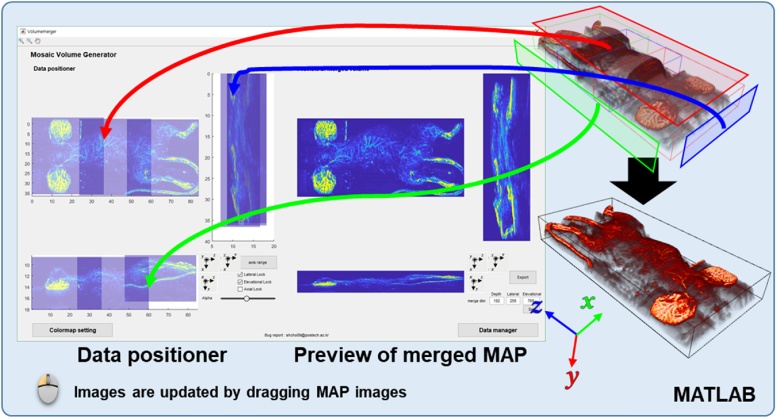
User interface of the “Mosaic volume generator” application (Supplementary Movie S1).

**Fig. 4:**
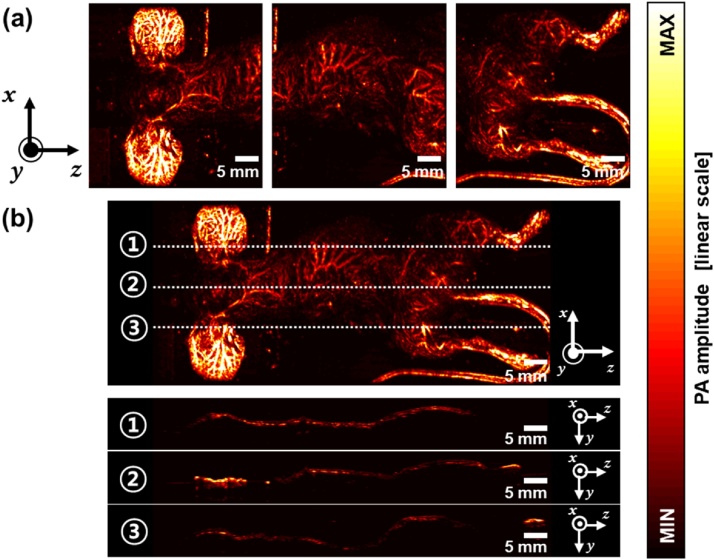
PA MAP mosaic image. (a) Three individual PA MAP input images. (b) PA MAP mosaic image and the depth-resolved PA B-scan images along the lines 1, 2, and 3. PA, photoacoustic and MAP, maximum amplitude projection.

The “Mosaic volume generator” application is shown in Fig. 3 and Supplementary Movie S1. The basic GUI consists of a data positioner, a merged images preview, a color map editor, a data manager, and window weighting. In this application, it is important to locate the individual volume data accurately. Thus, the interactive data positioner is designed to perform intuitive fine-tuning of the volume position. The data positioner has three different views for each orthogonal coordinate (i.e., x, y, and z axes). The data positioner allows the user to reposition each volume by dragging the PA maximum amplitude projection (MAP) images. The user can also set the volume location manually by entering an absolute location in the location editor. For fine-tuning the position, the user can take advantage of MATLAB's zoom and pan features. In the data positioner, the PA MAP images are displayed as translucent images to visualize overlapped areas. A 2D window weighting function is included in the data manager menu to perform appropriate window weight multiplication. In the merging process, zero padding and maximum intensity filtering are performed automatically, and the resulting MAP image in each orthogonal direction is displayed in the preview menu. After filtering, the merged volume could be exported from the preview menu. Fig. 4 shows the PA MAP mosaic image. Three individual PA MAP images (Fig. 4a) are merged into one seamless PA MAP mosaic image. The cross-sectional PA B-scan images are shown in Fig. 4b after seamless mosaic imaging.

### 3.2 Scan converter for optical scanning photoacoustic microscopy

#### 3.2.1 Background

**Fig. 5:**
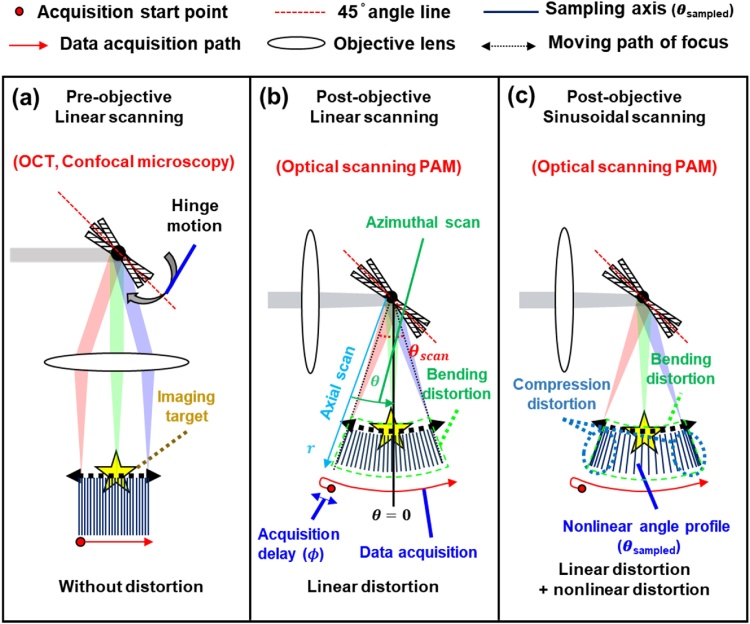
Image distortion vs various scanning methods.

The image distortions depending on various optical systems and scanning schemes and are shown in Fig. 5. The PAM images either suffer from linear distortion with linear scanning or linear and nonlinear distortions with sinusoidal scanning. The linear bending distortion is induced by the scanning geometry, and the nonlinear compression distortion is caused by the nonlinear movement of the scanning mirror. A pre-objective system that performs linear scanning shows no distortion in the acquired 3D image (Fig. 5a). However, image topology produced by a post-objective system with linear scanning (i.e., usually in optical scanning PAM [32]) is slightly deformed by a spherical (2D) or cylindrical (1D) pattern of light focusing on a target (Fig. 5b). The declination of the mirror causes additional distortions such as shear, scaling, and rotational deformation in the acquired images. As the azimuthal scanning geometry causes the linear distortion, this can be corrected through a linear transformation as follows [33]:

(1) $$\vec{p}=T\vec{r},$$

where $\vec{p}$ is a position vector in the Cartesian coordinator, T is a coordinator conversion matrix, and $\vec{r}$ is a position vector in the cylindrical or spherical coordinator. Furthermore, to remove the image distortion due to mirror declination, an affine transformation is applied as follows:

(2) $$\vec{p}=AT\vec{r},$$

where A is the affine transformation matrix. The 3D linear distortion can be effectively removed in optical scanning PAM with the linear scanning. More recently, a sinusoidal-driven galvanometer or MEMS mirrors have been applied to optical scanning PAM to achieve rapid scanning speeds via resonance motion [24,[34], [35], [36], [37], [38], [39], [40]]. When using a post-objective system with the sinusoidal scanning motion, the nonlinear distortion is also induced (Fig. 5c). In this case, the coordinator conversion and affine transformation cannot correct for distortion because of nonlinear distortion. Thus, the angle profile should be linearized through the following equation before correcting the distortion:

(3) $$\theta =\frac{\theta_{\mathrm{scan}}}{2}\text{sin}\left((\theta_{sampled}+\phi )\frac{\pi }{\theta_{scan}}\right),$$

where *θ_sampled_* is a sampled angle, *θ* is a linearized angle, *θ_scan_* is a scanning range of the scanner, and *ϕ* is a phase shift caused by acquisition delay. Without linearization, the images are divided into central and peripheral areas [41,42]. Since the difference between sin*θ* and *θ* are negligible in the central area, the angular interval between sampling axes is considered identical. However, the angular interval becomes dramatically nonlinear in the peripheral regions resulting expansion distortion. In the next section, we present a 3D scan converter for cylindrical coordination scanning with a sinusoidal mirror movement. We designed the 3D image warp transform based on inverse mapping approach to simultaneously remove linear and nonlinear distortions.

#### 3.2.2 Algorithm

**Fig. 6:**
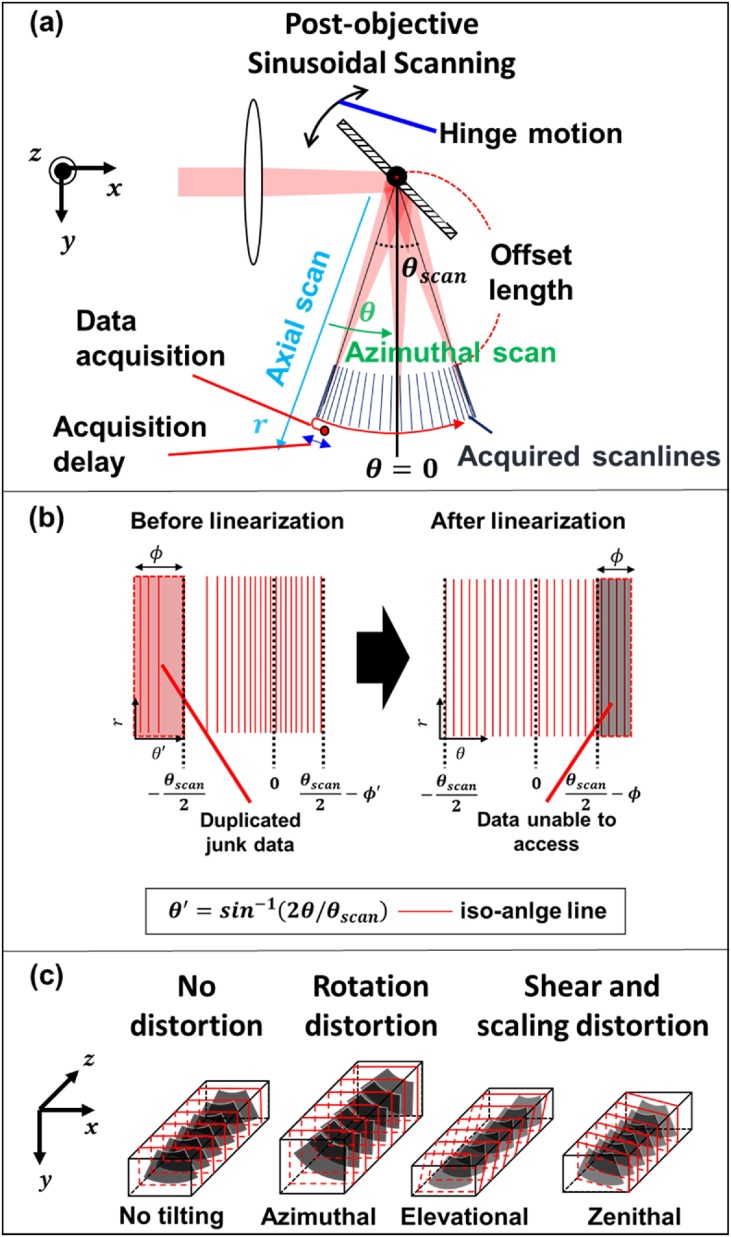
Principle of the scan conversion algorithm. (a) Scanning geometry and motion of the mirror. (b) Angular distribution of the acquired scanlines and angle profile linearization. (c) Effects of mirror declination in volumetric imaging.

As per the scanning mechanism in optical scanning PAM (Fig. 6a), the azimuth scanned data should be converted from the polar to Cartesian coordinator with consideration of declination and nonlinear motion of the mirror to correct the distortion. First, we perform the scan conversion by calculating the inverse representation of Eq. (2) as follows:

(4) $$\vec{r}=T^{-1}A^{-1}\vec{p}.$$

Since the elevational and zenithal tilting of the mirror induces shear deformation and the azimuthal tilting also brings rotational deformation, the affine matrix is represented as follows:

(5) $$A^{-1}=\left[\begin{array}{ccc} \text{cos}\delta_{azimuth} & 0 & -\text{sin}\delta_{azimuth}\\ 0 & 1 & 0\\ \text{sin}\delta_{azimuth} & 0 & \text{cos}\delta_{azimuth} \end{array}\right]\left[\begin{array}{ccc} \text{sec}\delta_{zenith} & 0 & 0\\ \text{tan}\delta_{zenith} & 1 & \text{tan}\delta_{elevation}\\ 0 & 0 & \text{sec}\delta_{elevation} \end{array}\right]$$

To remove this nonlinear distortion, the nonlinear angular profile between the scan lines are linearized by inverse mapping of the angle data (Fig. 6b) via the following Equation:

(6) $$\theta_{sampled}=\left({\text{sin}}^{-1}\frac{2\theta }{\theta_{scan}}\right)\frac{\theta_{scan}}{\pi }-\phi .$$

After the linearization, the 3D warping transformation is performed to remove the distortion in the 3D volume image (Fig. 6c). The 3D scan conversion algorithm then reduces both nonlinear and linear distortions, and is based on the following Equations:

(7) $$r=\left(\sqrt{{\left(x\text{sec}\delta_{zenith}\right)}^{2}+{\left(y\text{sec}\delta_{elevation}\right)}^{2}}-r_{\text{o}\text{f}\text{f}\text{s}\text{e}\text{t}}\right),$$

(8) $$\theta^{\text{'}}={\text{sin}}^{-1}\left(\frac{2\left({\text{tan}}^{-1}\left(\frac{x\text{cos}\delta_{elevation}}{y\text{cos}\delta_{zenith}}\right)-\delta_{azimuth}\right)}{\theta_{scan}}\right)\frac{\theta_{scan}}{\pi }-\phi ,$$

(9) $$z^{\text{'}}=z+x\text{tan}\delta_{zenith}+y\text{tan}\delta_{elevation},$$

where *x* (horizontal)*, y* (axial), and *z* (elevational) represent the location of the voxel to be restored in the Cartesian coordinator. The obtained data is being represented in the distorted cylindrical coordinate using the inverse sine scale angle instead of the normal angle. *r, θ'* and *z'* are the radial, distorted angle, and elevational positions, respectively. *ϕ* is the phase shift described in Fig. 6b. *δ* represent the declination of the mirror in each tilting direction in Fig. 6c.

#### 3.2.3 Application

**Fig. 7:**
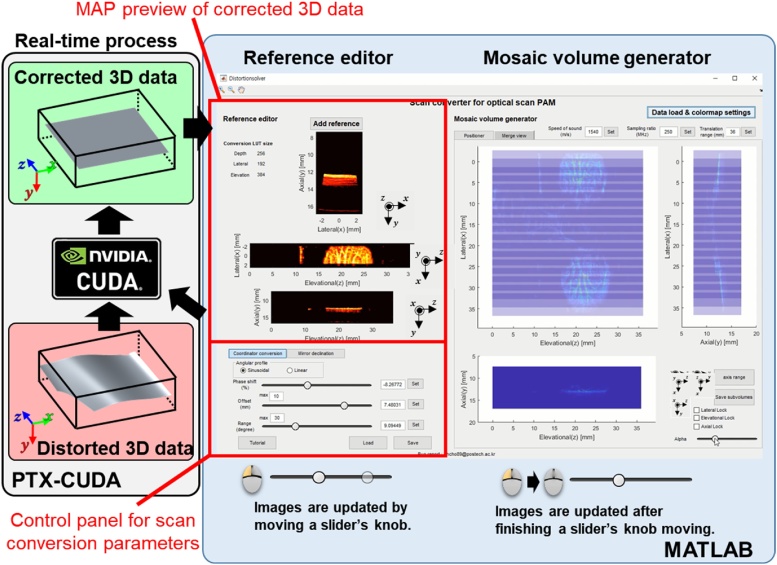
User interface of the “Scan converter for optical scanning photoacoustic microscopy” application (Supplementary Movie S2).

For an intuitive and interactive working environment, the volume scan conversions are carried out using CUDA GPGPU technology. The application supports binary files as input data with headers. Details of the file header are described in the user manual. To support binary file preparation, 3D PHOVIS provides class functions for adding headers and exporting binary files. Fig. 7 shows the screenshot of the “Scan converter” application, and the detailed process is shown in Supplementary Movie S2. The volume scan conversion is performed through two-step process. In the first step, “Reference editor” application chooses one data set as the reference volume to visualize the effects of real-time parameter adjustments, utilizing a GPU assisted scan conversion process. The following six parameters are interactively adjusted: (1) offset data radius, (2) scanning range, (3) azimuth tilt, (4) elevation tilt, (5) zenithal tilt, and (6) phase shift of mirror motion. In the second step, “Mosaic volume generator,” user confirms the adjusted parameters by comparing the relationship between different volume data sets. For these data sets, each image is updated after the mouse button from the parameter slider’s knob is released. Each loaded volume is visualized in “Data positioner” as a semi-transparent MAP image. By observing overlapped areas, the user can accurately optimize the parameters.

**Fig. 8:**
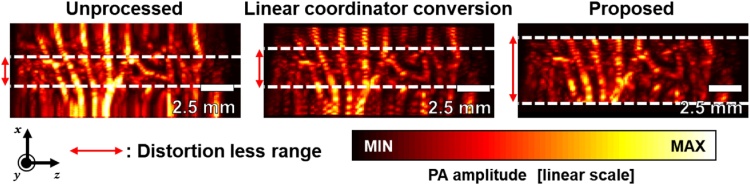
Comparison of the effective field of views in photoacoustic maximum amplitude projection images.

Without motion estimation, the area where *θ* ≒ sin^−1^*θ* is relatively free from distortion. Unlike the simple linear coordinator conversion method, the newly proposed scan conversion method achieves wider FOV and less distortion (Fig. 8). As one can see, the effective image length along the lateral direction increases to approximately 5 mm with our non-linear distortion correction, while they are about 2.5 mm in the linear coordinator conversion method and less than 2.5 mm in the unprocessed original image.

### 3.3 Skin profile estimator and depth encoder

#### 3.3.1 Background

Appropriate segmentation of the biological tissues is very important for 3D rendering. The depth-encoded colored PA MAP image is commonly used to show the 3D volumetric PA data in the 2D projection domains. However, a simple depth-encoded 3D rendered image based on the Cartesian coordinate induces significant errors in the rendered image because of irregular biological skin profiles. To accomplish successful depth encoding process, estimating the correct skin surface is essential [[43], [44], [45], [46]]. In the next sub-section, we build a software application that automatically assesses the skin profiles and creates the true colored depth-encoded images.

#### 3.3.2 Algorithm

Since the PA signals on the skin are not continuous, it is challenging to correctly contour the skin profiles in the PA images [45]. To delineate the continuous and smooth boundaries, the Gaussian blurring [46], cloth simulation [45], or random sample consensus (RANSAC) fitting [43] have been explored. While these methods show the convincing estimation of the skin profiles, they still have limitations. The Gaussian blurring and cloth simulation methods are vulnerable to outlier signals caused by artifacts. The RANSAC approach is robust even if the data contain many outliers, but only applicable when the appropriate analytical model exists.

**Fig. 9:**
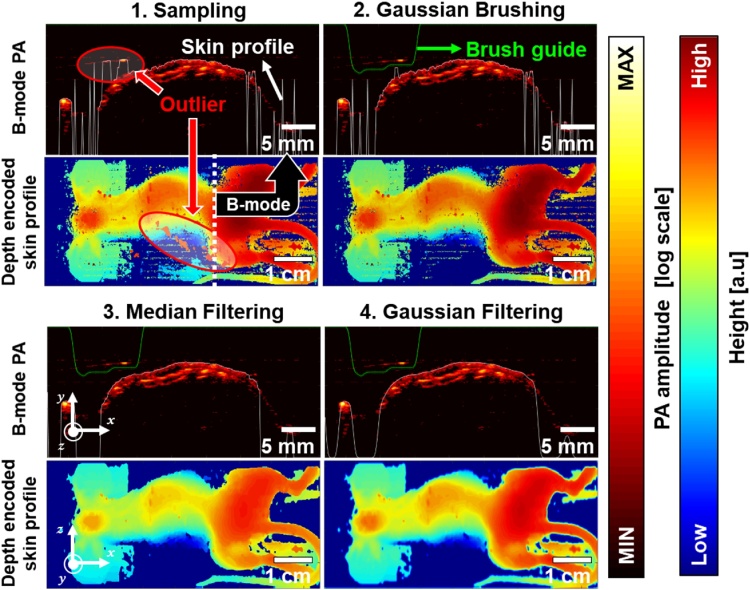
Skin profile estimation process in a B-mode photoacoustic (PA) image and estimated depth-encoded skin profile in the 3D PA image.

To estimate the skin profiles in the PA images, the rough skin profile is sampled by detecting the first PA signals along time-resolved A-line images. The sampled profile is filtered by median filtering and Gaussian blurring. The reliable skin profile, regardless of outliers, is achieved by manually tweaking the profile estimation process using the Gaussian Brush tool provided with the application. The detailed procedure is illustrated in Fig. 9. The white line indicates the estimated skin profile and the green line represents the brush guide. Typically, the outliers with large areas are excluded by the Gaussian Brushing and the smaller areas are removed by 2D median filtering. Finally, we apply the Gaussian filtering to smooth the profile.

#### 3.3.3 Application

**Fig. 10:**
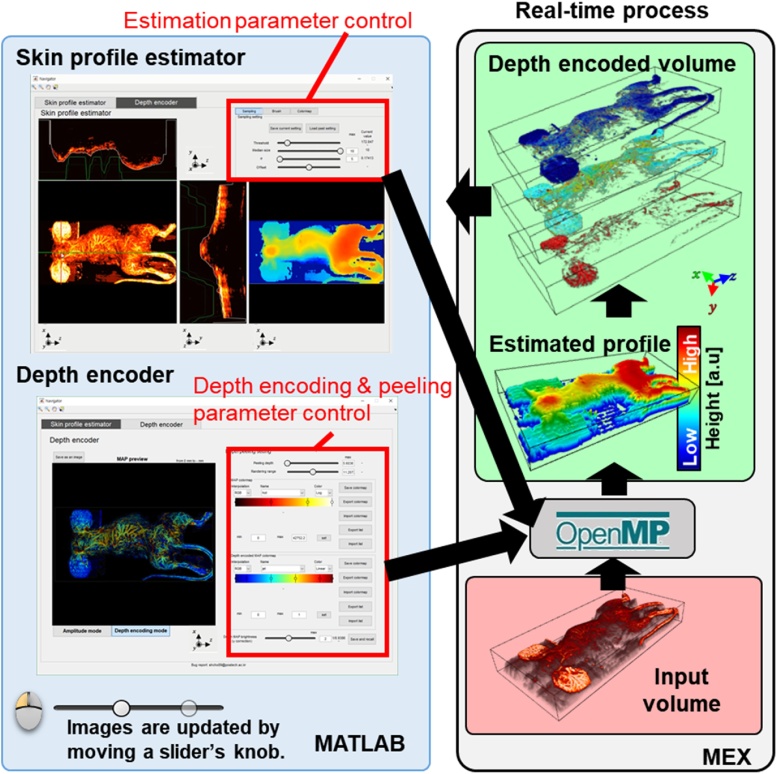
User interface of the “Skin profile estimator and depth encoder” application (Supplementary Movie S3).

The “Skin profile estimator and depth encoder” application estimates the skin profile and generates the colored depth-encoded image. This application is driven by MEX functions, which are parallelized by the OpenMP to interactively process and control the volume data in real-time. The GUI interface for this application is shown in Fig. 10, and the detailed process is shown in Supplementary Movie S3. The first panel is the “Skin profile estimator” panel. The profile estimation is controlled by four sliders. These slider control the sampling threshold, 2D square median filter size, Gaussian blur amount (using σ, standard deviation), and the overall offset movement of the estimated surface in the axial direction. The expected skin profile is visualized as a white line in each orthogonal slice image and projection image in the profile monitor. The Gaussian brush tool is provided to avoid any incorrect and noisy estimation. The Gaussian brush guides appear as green lines in each orthogonal slice image. The second panel is the “Depth encoder” panel. In this panel, regular and depth-encoded MAP images are displayed in the preview menu. All parameters for MAP rendering (e.g., a surface peel depth, a short-range MAP rendering range, rendering colors, and image brightness) are controlled by the depth peeling tool and two-color map editors. The depth peeling tool consists of two sliders that determine the depth of the surface peel and the range of rendering. The parameter settings of the peeling tool can be saved and recalled later by the reference manager. The signal brightness and depth encoding colors of MAP images are each controlled by separate color map editors. For additional brightness adjustment of the depth-encoded images, γ correction slider is provided. The generated MAP images, volume data, and depth indices can be exported from the Export menu.

### 3.4 3D volume viewer

#### 3.4.1 Background

Many 3D visualizations techniques have been developed and applied to visualize 3D PA data, and each has its own pros and cons [47]. Among various visualization methods, the multiplanar reconstruction (MPR) and volume rendering are most commonly used. The MPR generates a 2D image slice for any desired trajectory, and this method is easy and fast. The volume rendering generates a 3D-like image by simulating a virtual camera, volume subject, and light source. Since the visibility of the 3D image depends on the rendering algorithm and parameters, it is important to construct a specialized interactive interface to optimize the rendering algorithm and 3D parameter updates. To visualize 3D PA data effectively, we developed the "MPR viewer" and the "Volume renderer" with interactive interfaces in the MATLAB environment.

#### 3.4.2 Multiplanar reconstruction (MPR) viewer

##### 3.4.2.1 Algorithm

**Fig. 11:**
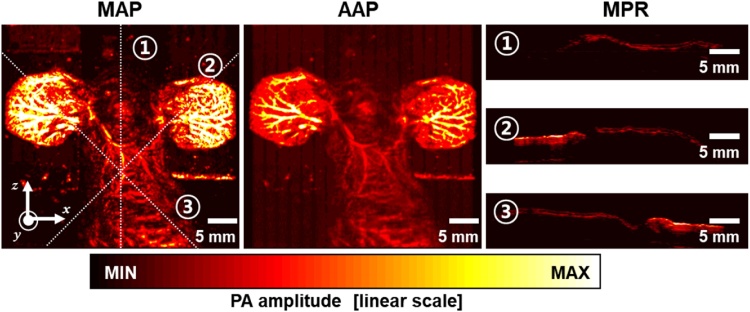
Maximum amplitude projection (MAP) image, average amplitude projection (AAP) image, and multiplanar reconstruction (MPR) images extracted along the lines “1”, “2”, and “3” in the MAP image.

We implement the MPR and projection rendering algorithms. Since each MPR slice is a radial slice in the viewing field, each 2D slice is reconstructed by the next linear transformation:

(10) $$\vec{r}=TR\vec{p},$$

where $\vec{r}$ is the position vector in the cylindrical coordinator, $\vec{p}$ is the position vector in the Cartesian coordinator, *T* is the coordination transform matrix, and *R* is the viewpoint rotation matrix. To provide navigation, the projection image is provided. The shear warp factorization algorithm [48] based on multi-core CPU and SSE is used to render the projection image in an arbitrary viewing direction. For the projection image, a MAP or average amplitude projection (AAP) image can be used. Fig. 11 shows one MAP image, one AAP image, and three MPR slices cut along the lines “1”, “2”, and “3” in the MAP image. Note that the navigation map image plays an important role in selecting the appropriate MPR configuration angle. In Fig. 11, the MAP image represents the distribution of high amplitude signals, while the AAP implies high-density signals.

##### 3.4.2.2 Application

**Fig. 12:**
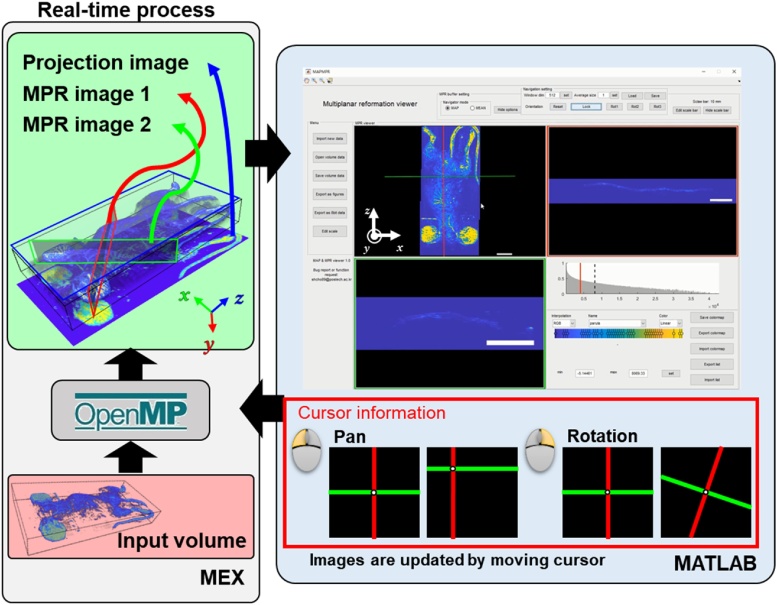
User interface of the “Multiplanar reformation viewer” application (Supplementary Movie S4). MPR: multiplanar reformation.

The user interface and control method of the MPR viewer application is shown in Fig. 12 and Supplementary Movie S4. The image reconstruction process is powered by the OpenMP library and by employing multi-core CPU parallelization. The MEX function reconstructs MPR, and projection images based on the input parameters, and imaging results are displayed in the GUI interface. The GUI interface has three parts: a navigation map, two MPR viewers, and a transfer function editor. The navigation cursor is controlled by two orthogonal lines with a center point in the projection map image. The position and angle of the cursor and the orthogonal lines are controlled by mouse movement. When the navigation cursor moves, the two MPR slices are automatically updated. The transfer function editor has interactive controls to instantly adjust the dynamic range and color map. The viewing direction in the navigation map can be rotated by right angle in each axial direction or in arbitrary direction. The arbitrary directional rotation is controlled by computer mouse motion.

#### 3.4.3 Volume renderer with a moviemaker

##### 3.4.3.1 Algorithm

**Fig. 13:**
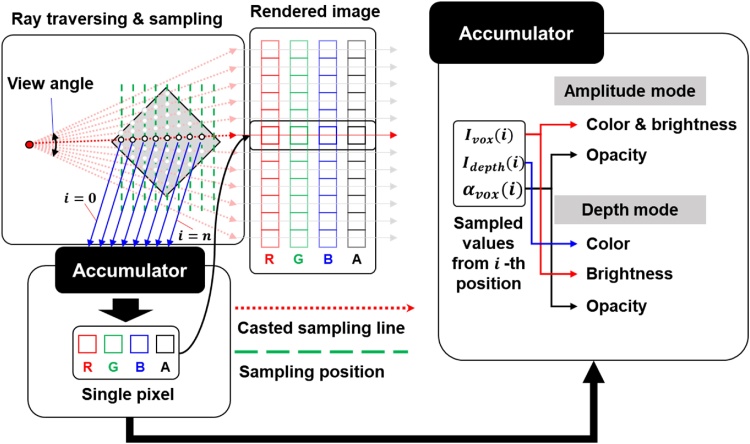
Principle of the volume ray casting process. *I_vox_*, the sampled PA signal amplitude value; *I_depth_*, the sampled PA imaging depth value; and *α_vox_*, the opacity of the sample.

We use a ray casting algorithm, which can provide the best image quality among volume rendering algorithms [49], for “Volume renderer” application. Because the volume rendering process is more computationally expensive than any other process, CUDA GPGPU acceleration is leveraged to execute the rendering processes in real-time. In volume rendering, it is important to determine the voxel information using opacity and colors [50]. We use colormaps to visualize the PA amplitudes in “Amplitude mode” and the PA depth information in “Depth mode.” To classify visible information, we simultaneously sample the PA amplitude and depth data. The detail rendering process is shown in Fig. 13 and it consists of a ray traversing, sampling, and accumulation process. The ray traversing process determines the sampling position depending on the traversing direction and sampling depth. We sample the values from the input data based on the calculated sampling position by trilinear interpolation. The accumulation process transfers the sampled values to red, green, blue and opacity values, and accumulates the sampled values to generate the rendered 2D images. In Fig. 13, *I_vox_*(*i*) is the sampled value that represents the PA signal amplitude, *I_depth_*(*i*) is the sampled imaging depth information of the volume, and *α_vox_*(*i*) is the opacity of the voxel data in the *i*-th sampling position. *I_vox_*(*i*) is the voxel’s color and brightness in the “Amplitude mode” and brightness in the “Depth mode”. *I_depth_*(*i*) is the voxel’s color in the “Depth mode”.

**Fig. 14:**
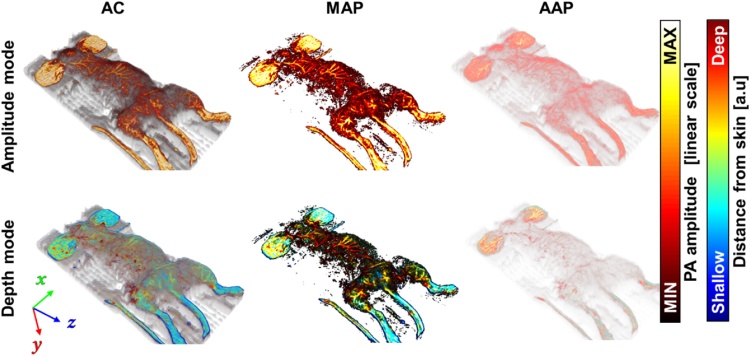
Photoacoustic amplitude and depth images processed through the AC, MAP, and AAP algorithms. AC: alpha composition, MAP: maximum amplitude projection, AAP: average amplitude projection.

Fig. 14 shows the rendering examples and corresponding algorithms in the “Amplitude mode”, and “Depth mode.” For the accumulation process, alpha composition (AC), MAP, and AAP methods are implemented. To render a single pixel, AC accumulates the sampled voxels with partial transparency [51], MAP finds the maximum values among the sampled voxels, and AAP returns the average values among the sample voxels along the corresponding transfer line. We used the depth index information generated from the “Skin profile estimator and depth encoder” application.

##### 3.4.3.2 Application

**Fig. 15:**
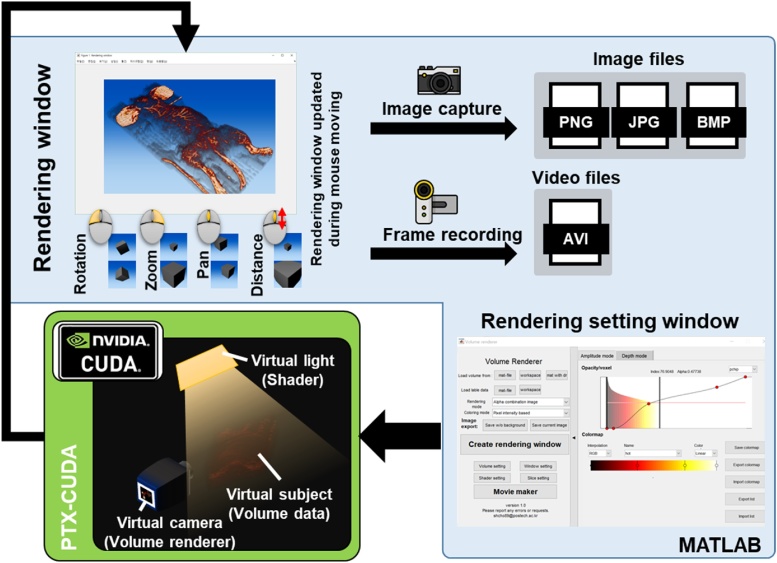
User interface of the “Volume renderer” application (Supplementary Movie S5). The example videos created by this application is available in Supplementary Movies S6–11.

The volume rendering application specializes in interactive real-time high-quality volume-rendered image and automatic motion frame generation. CUDA GPGPU acceleration technology is utilized for real-time volume rendering. Fig. 15 and Supplementary Movie S5 show the user interface for the “Volume renderer” application. For visualizing the volume data, the CUDA function generates a volume rendering image corresponding to the input data, and then the resulting image is displayed in the rendering window. The view orientation and perspective of an image can be fully adjusted in the render window with mouse control, while other software systems typically use a fixed distance between the camera and volume, or only supports non-perspective projection. The rendering results can be captured as an image file, or multiple image frames, which is generated by the automatic frame generator, can be recorded as a video file. The example videos created by this application is available in Supplementary Movies S6.

**Fig. 16:**
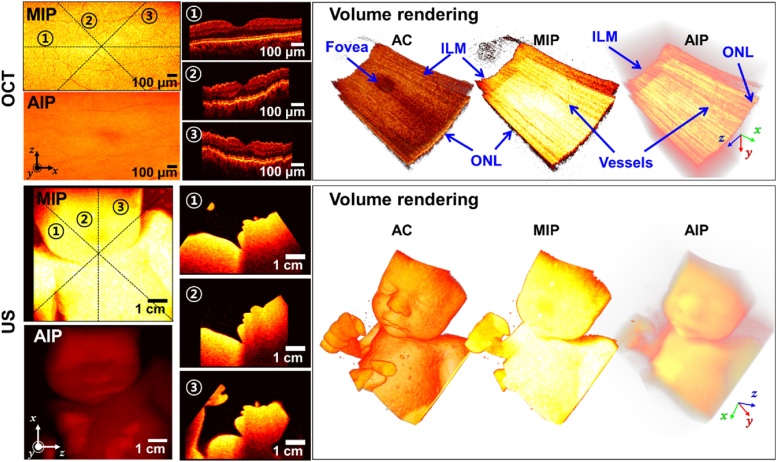
OCT and US MIP and AIP images and corresponding MPR images cut along the lines “1”, “2”, and “3” in the MIP images from “MPR viewer” and 3D volume rendered images processed by AC, MIP, and algorithms. The 3D volume rendered OCT and US images are shown in Supplementary Movie S10-11. AC: alpha composition, AIP: average intensity projection, MIP: maximum intensity projection, OCT: optical coherence tomography, US: ultrasound, ILM: inner limiting layer, ONL: outer nuclear layer.

We also explored the feasibility of our volume renderer for 3D OCT and US imaging. Fig. 16 shows the 2D projection, MPR images, and 3D volume-rendered OCT and US images. The volume-rendered movies are shown in Supplementary Movies S7-8. The OCT data published in Golabbakhsh et al. [25] is employed for OCT image rendering, and we acquired the 3D US volume data of a fetus phantom (Model 065-36, CISR Inc., Norfolk, VA) with a mechanically-swiveling 3D transducer interfaced to the EUB-6500 ultrasound machine (Hitachi Healthcare America, USA). The OCT and US maximum intensity projection (MIP, equivalent algorithm with MAP) and average intensity projection (AIP, an equivalent algorithm with AAP) images are based on optical and acoustic scattering, respectively. These examples imply that our volume renderer is flexible for use in various 3D volumetric imaging modalities.

## 4 System requirements

Software kits and user manuals are available on the Bio Optics and Acoustics Laboratory (BOA-Lab) website (www.boa-lab.com). All features are designed to work on 64-bit Windows platforms. All MEX functions have been compiled with a 64-bit compiler. Note that this software kit does not work with 32-bit MATLAB. MATLAB version 2016a or later is recommended for normal operation. The parallel and image processing toolboxes must be installed with MATLAB. Our software kit requires a resolution of 1920 × 1080 or higher for normal use. Otherwise, the application windows do not display images appropriately on the screen. The scan converter and volume renderer applications require a CUDA-capable video accelerator card. We further recommend using modern multi-core processors. 3D PHOVIS was developed and tested on workstations using Intel i5-4670 processor and NVIDIA GTX 750ti.

## 5 Discussion and conclusion

A variety of innovations have enhanced 3D PA imaging performance but ours is the first effort to enhance visualization of PA image, which is very critical for the analysis of results. Here, we demonstrated a specialized 3D visualization software package, 3D PHOVIS, specifically targeting photoacoustic data, image, and visualization processes. We incorporated 3D PHOVIS onto the MATLAB with graphical user interface and developed multi-core graphics processing unit modules for fast processing. The five main components of 3D PHOVIS are, namely mosaic volume generator, scan converter, skin profile estimator and depth encoder, MPR viewer, and volume renderer, have unique advantages. In the “Mosaic volume generator”, users provide flexibility to the user to intuitively and manually manipulate data volumes for better registration. Our maximum filtering method does not cause any signal loss or distortion, unlike other commonly used blending methods. Precise manual positioning and window weighting further minimizes data discrepancy for mosaic imaging [24]. The scan conversion algorithm proposed here corrects the distortion of nonlinear mirror scanning in PAM. The “Skin profile estimator and depth encoder” application provides a convenient depth peeling feature that allows us to visualize the unique characteristics of biological tissues based on depth information. The Gaussian brush tool in the “Skin profile estimator” can effectively correct false estimates caused by noises or artifacts. The intuitive slicing position control in the “MPR viewer” application is beneficial to analyze PA images and other 3D images like US and OCT images. In the “Volume renderer” application, three-volume rendering algorithms (e.g., AC, MAP, and AAP) shows their unique characteristics of volume data. As shown in Fig. 14, AC represents the most stereoscopic image, MAP represents the strong signal with a high contrast ratio, and AAP represents the signal density in the viewing direction. Since, we implemented the 3D PHOVIS on the most commonly used MATLAB platform, it provides researchers to incorporate their own newly developed visualization algorithm to improve the image quality further. 3D PHOVIS can directly import any type of data from the MATLAB workspace. In addition, we also demonstrated the applicability of this software to ultrasound imaging and optical coherence tomography. User manuals and application files for 3D PHOVIS are available for free on the website (www.boa-lab.com). We believe our 3D PHOVIS provides a unique tool to PAI researchers, expedites its growth, and attracts broad interests in a wide range of studies. In addition, our 3D software has several advantages over other commercially available including it is free, the applications available are optimized for PA imaging, and is based upon most commonly used research platform, i.e., MATLAB.

There are some limitations to 3D PHOVIS, which can be overcome in near future. The proposed scan conversion algorithm only works for 1-axis cylindrical scanning and different algorithms exists for 2-axes mirror scanning [35] or other scanning mechanisms, which can be incorporated into 3D PHOVIS. Some modules are operator dependent, such as “Mosaic volume generator” and “Skin profile estimator and depth encoder” applications. Future research can automate these applications and incorporated into 3D PHOVIS for other researchers to use. In addition, since all the features and class methods of 3D PHOVIS are designed for GUI input, they are not configured properly for script coding without GUI control. More sophistication can be added to the GUI control. Nevertheless, we strongly believe the proposed 3D visualization tool is very valuable for high-quality research activities in the field of biological and biomedical imaging.

## Declaration of Competing Interest

Chulhong Kim has financial interests in OPTICHO, which, however, did not support this work.
